# Diagnostic nomogram based on ultrasound and clinical data of predicting malignant lymph nodes in HIV patients with lymphadenopathy

**DOI:** 10.3389/fcimb.2025.1622903

**Published:** 2025-09-08

**Authors:** Lin Pan, Chaoting Yang, Huaguo Shao

**Affiliations:** ^1^ Department of Ultrasound, Hangzhou Xixi Hospital Affiliated to Zhejiang Chinese Medical University, Hangzhou, Zhejiang, China; ^2^ The Fourth Clinical Medical College, Zhejiang Chinese Medical University, Hangzhou, Zhejiang, China; ^3^ Clinical Research Laboratory, Hangzhou Xixi Hospital Affiliated to Zhejiang Chinese Medical University, Hangzhou, Zhejiang, China

**Keywords:** HIV, ultrasound, T cell subsets, diagnosis, malignancy

## Abstract

**Background and aims:**

Acquired Immune Deficiency Syndrome (AIDS), caused by Human Immunodeficiency Virus (HIV), leads to severe immunodeficiency, making patients susceptible to opportunistic infections and malignancies. Lymphadenopathy is a common symptom in AIDS patients, reflecting immune system responses but also indicating potential disease progression. Distinguishing between benign and malignant lymphadenopathy is crucial for appropriate treatment. This study aimed to develop a diagnostic method for differentiating benign and malignant lymph nodes in HIV-infected patients using clinical and ultrasound data.

**Methods:**

The study was conducted at Hangzhou Xixi Hospital from March 2016 to March 2024, including 149 HIV patients with confirmed lymphadenopathy. Ultrasound examinations were performed to assess lymph node characteristics, and biopsies were conducted for pathological confirmation. Statistical analysis involved the least absolute shrinkage and selection operator (LASSO) regression to identify significant predictors and construct a nomogram for predicting lymph node malignancy.

**Results:**

The malignant lymph nodes had larger short and long diameters, and differences in shape, echogenicity, and hilum compared to benign lymph nodes. Lymphocyte count and T cell subsets were higher in malignant lymph nodes. The LASSO regression model identified short diameter, lymphocyte ratio, CD3^+^ T cell count, and CD4^+^ T cell ratio as significant predictors. The nomogram constructed based on these features demonstrated good predictive accuracy (AUC = 0.904).

**Conclusions:**

In conclusion, our study developed a diagnostic nomogram based on clinical and ultrasound data to differentiate benign and malignant lymph nodes in HIV patients. This tool had diagnostic accuracy and offers practical guidance for clinical management of HIV patients with lymphadenopathy.

## Introduction

Acquired Immune Deficiency Syndrome (AIDS) is a severe immunodeficiency disease caused by infection with the Human Immunodeficiency Virus (HIV) (Gallo and Montagnier, 2003). HIV primarily targets CD4^+^ T cells, leading to a significant reduction in their numbers and a gradual decline in immune function (Duesberg, 1993; Aikman, 1994; Jocelyn et al., 2024). As the disease progresses, patients become more susceptible to various pathogens, making them prone to opportunistic infections such as pneumocystis pneumonia, tuberculosis, cytomegalovirus infection, and malignant tumors (Arribas et al., 1996; Cribbs et al., 2020; Lurain et al., 2024). HIV binds to lymphocytes, disrupting their normal function and causing uncontrolled proliferation of lymphocytes and disorganization of lymph node structure, resulting in lymphadenopathy (Malin et al., 1994; Mudd et al., 2013).

In the clinical manifestations of AIDS, lymphadenopathy is one of the most common and important symptoms (Banga et al., 2021). It not only reflects the immune system’s response to pathogens but may also indicate the progression of the disease and potential complications (Tenner-Racz et al., 1988). lymphadenopathy is a defensive reaction of the immune system to pathogens or abnormal cells, however, it may have adverse effects on the body if persists or progresses (Lederman and Margolis, 2008; Fletcher et al., 2022). For example, enlarged lymph nodes can cause local compression symptoms, such as limb swelling due to vascular compression and pain or sensory abnormalities due to nerve compression (Dianzani et al., 1996). Moreover, lymphadenopathy can be a sign of underlying diseases, such as infections or tumors. If left untreated, it may lead to further disease progression (Dimopoulos et al., 2017).

Benign lymphadenopathy is usually a normal response of the body to infection or inflammation (Brousset et al., 1996). After appropriate anti-infective or anti-inflammatory treatment, the lymph nodes can return to normal. However, malignant lymphadenopathy is often a sign of tumors, such as lymphoma or metastatic tumors, which require timely targeted treatments, including chemotherapy, radiotherapy, and surgical resection (Rosen et al., 2022; Antel et al., 2023). Misdiagnosing malignant lymphadenopathy as benign can delay treatment, leading to further tumor spread and deterioration, which can severely affect the patient’s prognosis and survival rate (Cainelli et al., 2010). Distinguishing between benign and malignant lymphadenopathy is of great importance for clinical diagnosis and treatment (Stein et al., 2016; Sanchez-Cabral et al., 2025). Ultrasound is a non-invasive, convenient, and highly repeatable imaging method that has significant advantages in differentiating benign and malignant lymphadenopathy (Obajimi et al., 2008; Sculier et al., 2010). It can clearly display the size, shape, internal structure, and blood flow of lymph nodes, providing important evidence for clinical diagnosis.

Therefore, this study aimed to analyze the clinical and ultrasound data of patients, screen for classification features, and construct a stable and convenient diagnostic method for differentiating benign and malignant lymph nodes.

## Methods

### Patients

This study was a retrospective observational study conducted at Hangzhou Xixi Hospital. From March 2016 to March 2024, patients with HIV who were admitted to our hospital and underwent ultrasound examination with confirmed superficial lymphadenopathy were included in this study. Inclusion criteria were as follows: (1) a definitive diagnosis of AIDS; (2) ultrasound examination with confirmed lymphadenopathy; (3) pathological results from biopsy of the enlarged lymph nodes. Exclusion criteria were as follows: (1) inadequate or insufficient tissue obtained from lymph node biopsy, making pathological diagnosis impossible; (2) patients under 18 years of age; (3) non-standard ultrasound images in the routine examination, lacking color Doppler images or with poor image quality. This study was approved by the Clinical Research Ethics Committee of the Hangzhou Xixi Hospital.

### Ultrasound examination

A GE LOGIQ E9 color Doppler ultrasound diagnostic instrument with a linear array probe (frequency range: 7.0–12.0 MHz) was used. Routine scanning of the patient’s neck, axilla, and inguinal regions was performed to observe the size, shape, internal echogenicity, borders, calcifications, cystic changes, lymph node hilum, presence of edema in surrounding tissues, and blood flow signals of the enlarged lymph nodes. When scanning cervical lymph nodes, position the patient supine and place a pillow under the neck to fully expose the area. For examination of one side, have the patient turn the head to the opposite side and follow the seven anatomical levels of head-and-neck nodal regions in sequence. For the axillary nodes, ask the patient to abduct the arm overhead to expose the axilla fully. Place the transducer on the medial aspect of the upper arm and slide it along the axillary vein toward the apex to visualize the lateral and apical nodes, then move medially along the axillary wall to assess the central nodes. For the inguinal region, instruct the patient to extend and slightly separate the legs to expose the groin. Scan transversely and longitudinally along the inguinal ligament and great saphenous vein, proceeding from superior to inferior and from lateral to medial. When an enlarged target node is identified, use the minimal pressure that still reveals its internal architecture to avoid compression artifacts that would falsify measurements or eliminate flow signals. Save the longitudinal and transverse images of the node at its largest cross-section, together with a color doppler flow imaging (CDFI) image. If calcifications, cystic changes, or other special features are present, capture an additional image that highlights these characteristics. In CDFI mode, pay particular attention to the pattern of intranodal blood flow. Ultrasound images were captured and stored in real time. The images were analyzed jointly by a deputy chief physician and a physician with over five years of experience in the department.

For ultrasound guided puncture, a disposable biopsy needle produced by Bard was used. Under real-time ultrasound guidance, the disposable biopsy needle was inserted into the target lymph node to obtain two strips of white tissue, each approximately 15 mm in length. The tissue samples were placed in a fixative solution and sent to the pathology department for histopathological examination, including periodic acid–schiff (PAS) staining and hexamine silver staining. Some samples underwent immunohistochemical testing.

### Statistical analysis

For quantitative variables, the quartiles, minimums and maximums were used to describe the central tendency and dispersion of the data. The non-parametric wilcoxon rank-sum test was applied to compare differences between quantitative variables. For qualitative data, we described the data using frequencies and proportions. Comparisons between categorical variables were performed using the Chi-square test or Fisher’s exact test. Fisher’s exact test was preferred when the sample size was small or the expected frequency in any cell was less than five.

To identify significant predictors, we employed the least absolute shrinkage and selection operator (LASSO) regression model to reduce overfitting and select the most relevant predictors from a large pool of variables. The optimal regularization parameter was determined using cross-validation to balance model complexity and predictive accuracy.

To address the issue of class imbalance in our dataset, we employed the synthetic minority over-sampling technique (SMOTE) to generate synthetic samples for the minority class, thereby balancing the dataset and improving model performance. Specifically, we used the SMOTE function from the DMwR package with the following parameters: perc.over = 500 (doubling the minority class), perc.under = 120 (randomly under-sampling the majority class to match the augmented minority size) and other parameters were default. This approach ensured a 1:1 class ratio in the generated data.

For evaluating the predictive performance of the models, we utilized the receiver operating characteristic (ROC) analysis. The area under the ROC curve (AUC) was calculated to quantify the overall predictive accuracy, with higher AUC values indicating better discrimination ability. An AUC of 0.5 suggested no discrimination ability, while an AUC of 1.0 indicated perfect discrimination.

Additionally, we constructed a nomogram based on the final multivariate model to provide a visual tool for predicting the probability of the outcome. A nomogram is a graphical representation that integrates multiple predictors into a single score, allowing for easy and intuitive interpretation of the model’s predictions. The nomogram was calibrated and validated using bootstrapping techniques to ensure its accuracy and reliability in clinical practice.

In statistical methods, a *P* value < 0.05 was considered statistically significant. All analyses were conducted by R version 4.4.2.

## Results

### Characteristics of patients

A total of 149 HIV patients with lymphadenopathy were enrolled after screening by inclusion and exclusion criteria, comprising 19 females and 130 males (**Table 1**). Among them, lymph node tissues from 18 patients (12.1%) were confirmed malignant after pathological examination with a median age of 47.5 years (min: 26 years, Q1: 41 years, Q3: 58 years, max: 82 years), while 131 patients with benign lymph nodes (*P* = 0.081) had a median age of 43 years (min: 20 years, Q1: 31 years, Q3: 53 years, max: 77 years). Malignant lymph nodes had longer short diameter (malignant: median 1.6 mm, benign: median 1.1 mm, *P* < 0.001) and long diameter (malignant: median 3.25 mm, benign: median 2.4 mm, *P* = 0.009) than benign lymph nodes. Characteristics of ultrasound examination showed differences between malignant and benign lymph nodes in shape (*P* = 0.035), echogenicity (*P* = 0.025) and hilum (*P* = 0.006). In the results of T cell subsets, malignant lymph nodes had higher values in lymphocyte count (*P* < 0.001), CD3^+^ T cell count(*P* < 0.001), CD4^+^ T cell count(*P* < 0.001), CD4^+^ T cell ratio (*P* < 0.001), B cell count (*P* = 0.014), CD8^+^ T cell count (*P* = 0.003), NK cell count (*P* = 0.017) and CD4^+^/CD8^+^ T cell ratio (*P* = 0.001).

**Table 1:** Clinical and ultrasound characteristics of patients.

Characteristic	Overall (N = 149)	Benign (N = 131)	Malignant (N = 18)	*P*-value
Gender				>0.999
Female	19 (13%)	17 (13%)	2 (11%)	
Male	130 (87%)	114 (87%)	16 (89%)	
Age (years)				0.081
median (Q1,Q3)	43.00 (32.00,53.00)	40.00 (31.00,53.00)	47.50 (41.00,58.00)	
min,max	20.00,82.00	20.00,77.00	26.00,82.00	
Long diameter (mm)				0.009
median (Q1,Q3)	2.50 (2.00,3.20)	2.40 (2.00,3.10)	3.25 (2.50,4.30)	
min,max	1.20,12.00	1.20,4.60	1.50,12.00	
Short diameter (mm)				<0.001
median (Q1,Q3)	1.10 (0.90,1.50)	1.10 (0.80,1.50)	1.60 (1.10,2.90)	
min,max	0.40,7.70	0.40,2.90	0.70,7.70	
Shape				0.035
Irregular	43 (29%)	34 (26%)	9 (50%)	
Regular	106 (71%)	97 (74%)	9 (50%)	
Echogenicity				0.025
Hyperechoic	28 (19%)	28 (21%)	0 (0%)	
Hypoechoic	121 (81%)	103 (79%)	18 (100%)	
Border				0.599
Defined	142 (95%)	124 (95%)	18 (100%)	
Undefined	7 (4.7%)	7 (5.3%)	0 (0%)	
Calcification	1 (0.7%)	1 (0.8%)	0 (0%)	>0.999
Hilum				0.006
Absent	72 (48%)	57 (44%)	15 (83%)	
Present	57 (38%)	55 (42%)	2 (11%)	
Thinned	20 (13%)	19 (15%)	1 (5.6%)	
Cystic degeneration	28 (19%)	26 (20%)	2 (11%)	0.527
Edema	39 (26%)	37 (28%)	2 (11%)	0.158
Blood flow signal				0.098
Abundant	81 (54%)	67 (51%)	14 (78%)	
No	15 (10%)	14 (11%)	1 (5.6%)	
Poor	53 (36%)	50 (38%)	3 (17%)	
Lymphocyte count				<0.001
median (Q1,Q3)	770.00 (390.00,1,380.00)	730.00 (350.00,1,330.00)	1,345.00 (920.00,1,920.00)	
min,max	50.00,5,270.00	50.00,2,920.00	520.00,5,270.00	
Lymphocyte ratio				0.152
median (Q1,Q3)	17.40 (10.20,25.40)	16.50 (9.60,25.40)	20.15 (15.00,26.70)	
min,max	1.80,80.20	1.80,80.20	7.60,44.50	
CD3^+^ T cell count				<0.001
median (Q1,Q3)	580.00 (320.00,1,047.00)	525.00 (260.00,910.00)	1,053.50 (659.00,1,375.00)	
min,max	7.80,3,931.00	7.80,2,351.00	422.00,3,931.00	
CD3^+^ T cell ratio				0.679
median (Q1,Q3)	77.90 (64.00,83.10)	78.00 (63.20,83.10)	75.85 (68.70,85.50)	
min,max	43.40,95.50	43.40,95.50	61.00,91.40	
CD4^+^ T cell count				<0.001
median (Q1,Q3)	86.00 (21.00,211.00)	72.00 (17.00,185.00)	267.00 (126.00,384.00)	
min,max	0.00,2,461.00	0.00,1,218.00	81.00,2,461.00	
CD4^+^ T cell ratio				<0.001
median (Q1,Q3)	10.70 (4.50,20.00)	9.90 (3.50,17.80)	21.10 (15.20,30.60)	
min,max	0.20,49.70	0.20,49.70	4.80,46.70	
B cell count				0.014
median (Q1,Q3)	59.00 (19.00,144.00)	55.00 (18.00,136.00)	125.00 (50.00,206.00)	
min,max	1.00,590.00	1.00,451.00	12.00,590.00	
B cell ratio				0.827
median (Q1,Q3)	8.00 (4.10,13.40)	7.90 (3.90,13.40)	9.55 (4.40,13.40)	
min,max	0.20,48.50	0.20,48.50	1.20,30.10	
CD8^+^ T cell count				0.003
median (Q1,Q3)	377.00 (223.00,669.00)	361.00 (210.00,658.00)	655.00 (364.00,968.00)	
min,max	17.00,2,726.00	17.00,1,852.00	297.00,2,726.00	
CD8^+^ T cell ratio				0.128
median (Q1,Q3)	57.70 (42.60,68.50)	58.40 (43.60,68.80)	46.05 (37.60,62.30)	
min,max	10.60,85.10	10.60,85.10	27.50,81.50	
NK cell count				0.017
median (Q1,Q3)	79.00 (45.00,184.00)	72.00 (42.00,182.00)	161.50 (62.00,323.00)	
min,max	8.00,890.00	8.00,890.00	39.00,696.00	
NK cell ratio				0.344
median (Q1,Q3)	11.60 (7.80,19.50)	11.70 (8.00,20.30)	9.40 (7.00,17.00)	
min,max	2.00,149.00	2.00,149.00	2.70,29.90	
CD4^+^/CD8^+^ T cell ratio				0.001
median (Q1,Q3)	0.18 (0.07,0.44)	0.16 (0.06,0.34)	0.42 (0.27,0.85)	
min,max	0.00,4.69	0.00,4.69	0.06,1.30	

### LASSO regression model

All 25 characteristics in **Table 1** were used to build a LASSO regression model. Because of the small malignant samples, the SMOTE method was conducted to achieve a 1:1 ratio between the two groups of samples. There were 108 samples in each group after over-sampling. Samples were divided into one training set and one test set randomly in a 7:3 ratio.

**Figure 1:**
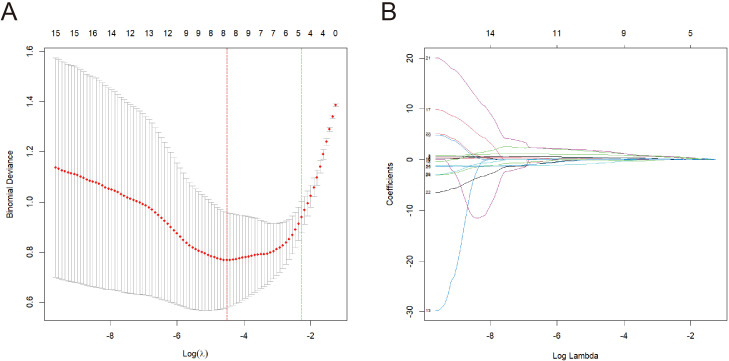
Selection of features and optimal parameter λ of LASSO regression model. **(A)** Cross-validation curve. **(B)** The relationship between coefficient and λ.

The LASSO regression model identifies the optimal parameter λ and features through ten-fold cross-validation. In **Figure 1A**, the red line indicated the value of λ at which the mean error is minimized, and the model achieved the best fit (λ = 0.011). The blue line indicated the largest value of λ within one standard deviation of the mean error, which also provided a good model fit but with fewer selected features (λ = 0.102). **Figure 1B** showed the relationship between λ and the coefficients of the features. Based on the ranking of the coefficients of the features and the number of features corresponding to the optimal λ, the top four features were selected, which were short diameter, lymphocyte ratio, CD3^+^ T cell count, and CD4^+^ T cell ratio.

**Figure 2:**
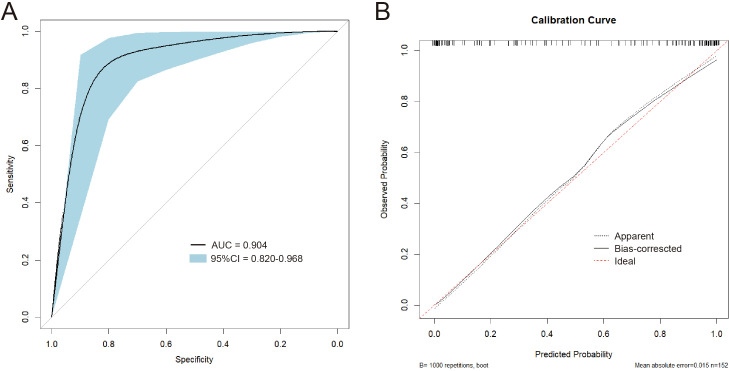
**(A)** The ROC curve of the LASSO model validated on the test set. **(B)** The calibration curves of the nomogram.

The LASSO regression model was subsequently validated in the test set. **Figure 2A** showed the AUC was 0.904 (**Figure 2A**). Furthermore, the accuracy, sensitivity, specificity, positive predictive rate, and negative predictive rate were 0.875, 0.938, 0.813, 0.833, and 0.929, respectively.

### Plotting of nomogram

**Figure 3:**
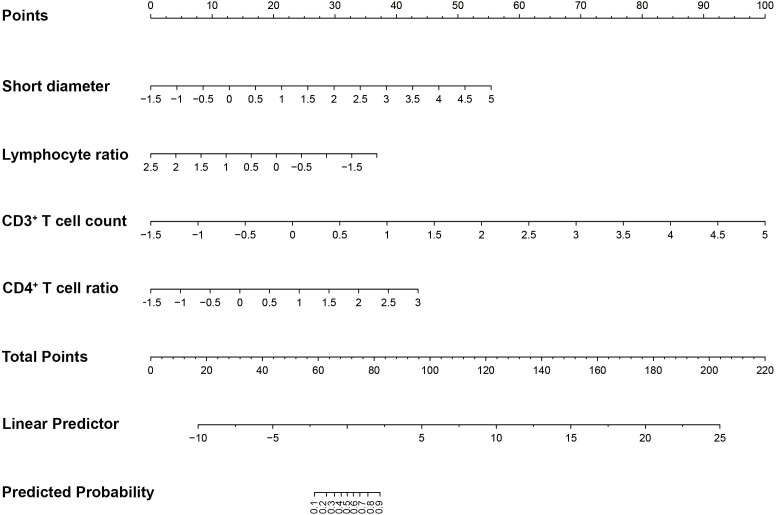
The nomogram for predicting the malignancy of the swollen lymph nodes in HIV patients.

To plot the nomogram, a logistic regression model was built. In **Figure 2B**, the calibration curves of the nomogram indicated that predictive results of the model were reliable and the calibration performance was good. The nomogram for predicting the malignancy of the swollen lymph nodes in HIV patients was constructed based on data of the short diameter, lymphocyte ratio, CD3^+^ T cell count, and CD4^+^ T cell ratio from training set (**Figure 3**). For each test result of a patient, a vertical line was drawn upward to intersect with the score line segment, obtaining the score for that indicator. Then, the scores of all indicators were added together to get the total score. Finally, a vertical line was drawn downward from the total score axis to determine the probability of the patient’s lymph node being diagnosed as malignant.

## Discussion

In summary, we prospectively analyzed 149 HIV-positive patients with lymphadenopathy and identified several distinguishing features between benign and malignant nodes. Malignant lymph nodes exhibited longer short and long diameters, with distinct ultrasound characteristics such as shape, echogenicity, and hilum visibility. In addition, malignant cases displayed higher total lymphocyte counts and altered T-cell subset distributions. Utilizing LASSO regression, we identified four key predictors: short diameter, lymphocyte ratio, CD3^+^ T cell count, and CD4^+^ T cell ratio. The nomogram constructed from these predictors demonstrated robust predictive performance, with an AUC of 0.904 and good calibration.

The proposed model incorporating short diameter, lymphocyte ratio, CD3^+^ T cell count, and CD4^+^ T cell ratio demonstrates significant potential in differentiating benign from malignant lymphadenopathy in HIV patients. These parameters reflect key immunological and structural changes associated with HIV progression and lymph node pathology. The lymphocyte ratio functions as an indirect gauge of systemic immune competence (Lane et al., 2005). HIV primarily targets CD4^+^ T cells, which is a well-known prognostic marker in HIV (Mellors et al., 1997). A declining CD4^+^ T cell ratio associates with severe immunosuppression, increasing vulnerability to malignancies. CD3^+^ T cell count reflects overall T-cell immunity. HIV-induced depletion of CD3^+^ cells disrupt immune surveillance, facilitating oncogenic viral infections that drive lymphomagenesis (Schellekens et al., 1990). Short diameter is a key morphological indicator. Malignant lymphadenopathy, such as lymphoma or metastatic disease, often presents with larger and more irregular nodal dimensions compared to reactive lymphadenopathy. In HIV infection, persistent lymph node enlargement may signal opportunistic infections or neoplasms, necessitating differentiation via imaging and histopathology.

In this study, patients with malignant lymph nodes exhibited higher CD4^+^ T cell counts and a higher CD4^+^/CD8^+^ T cell ratio than those with benign nodes, which appears counterintuitive. These findings may be due to several causes such as immune-microenvironment remodeling or small-sample bias. Malignant lymph nodes can secrete abundant cytokines that locally stimulate T cell proliferation and infiltration, thereby increasing the absolute number of CD4^+^ T cells. Additionally, our malignant cohort was small, introducing potential selection bias that could deviate from the true distribution in the broader HIV-infected population.

Recent advances in multimodal diagnostic approaches have demonstrated significant clinical value across various medical specialties. Wang et al. combined ultrasonography with serum CA19–9 and CEA to create a nomogram that accurately predicts malignant transformation of cystic lesions (Wang et al., 2022). Similarly, Bjurlin and colleagues developed and validated an MRI and ultrasound fusion-guided nomogram that effectively stratifies prostate cancer risk following negative biopsies, demonstrating the power of combining advanced imaging with clinical parameters (Bjurlin et al., 2019). Unlike the pancreatic model that relies on nonspecific tumor markers, our approach incorporates HIV-specific immunological parameters including CD3^+^ and CD4^+^ T cell counts, which directly reflect the underlying pathophysiological mechanisms of lymphomagenesis in immunodeficient hosts. The diagnostic performance of our model compares favorably with these established systems, while maintaining greater clinical accessibility through the exclusive use of conventional ultrasound and routine laboratory tests.

The present study has several limitations that may affect the generalizability and accuracy of the results. First, this is a single-center study, with all data collected from the same medical institution. This study design may limit the external validity of the findings, as medical conditions, patient population characteristics, and diagnostic criteria may vary across different regions. Second, the number of positive samples in this study is relatively small, accounting for less than 20% of the total sample size, which increases the risk of small-sample bias and overfitting. This imbalance in sample proportion may lead to biases in statistical analysis, restrict in-depth analysis of the characteristics of malignant lymph nodes, and reduce the robustness of the study results. Future studies should aim to increase the number of positive samples to enhance the reliability and representativeness of the findings. In addition, SMOTE assumed that the feature space between existing minority-class cases is linear and evenly distributed; this assumption may not hold in complex immunological or imaging data, potentially distorting decision boundaries. Future work must therefore include external validation using larger, multicenter datasets and alternative resampling strategies to confirm the robustness and generalizability of our findings. Finally, there are differences in patient laboratory test information and ultrasound examination information among different hospitals and doctors. Disparities in equipment, protocols, and operator expertise can yield heterogeneous results. These differences may affect the accuracy and comparability of the data, thereby potentially impacting the reliability of the study results. To minimize such biases, future studies could consider a multicenter design to ensure the consistency and standardization of data collection.

## Conclusion

In conclusion, our study developed a diagnostic nomogram based on clinical and ultrasound data to differentiate benign and malignant lymph nodes in HIV patients. This tool had diagnostic accuracy and offers practical guidance for clinical management of HIV patients with lymphadenopathy.

## Data Availability

The data supporting the conclusions of this article will be available from the corresponding author on reasonable request. Requests to access these datasets should be directed to HS, shaohuaguocn@outlook.com.
